# Core components of male-specific person-centred HIV care: a qualitative analysis from client and healthcare worker perspectives in Malawi

**DOI:** 10.1136/bmjph-2024-001100

**Published:** 2024-12-22

**Authors:** Julie Hubbard, Misheck Mphande, Isabella Robson, Kelvin Balakasi, Khumbo Phiri, Elijah Chikuse, Marguerite Thorp, Sam Phiri, Augustine T Choko, Morna Cornell, Thomas Coates, Kathryn Dovel

**Affiliations:** 1 Division of Infectious Diseases, David Geffen School of Medicine, University of California Los Angeles, Los Angeles, California, USA; 2 Implementation Science Department, Partners in Hope Medical Center, Lilongwe, Central Region, Malawi; 3 Malawi Liverpool Wellcome Trust, Blantyre, Malawi; 4 Centre for Infectious Disease Epidemiology & Research, School of Public Health, University of Cape Town, Cape Town, South Africa; 5 University of California Global Health Institute, San Francisco, California, USA

**Keywords:** HIV, Community Health, Communication, Education, Medical

## Abstract

**Introduction:**

Person-centred care (PCC) improves clinical outcomes for people living with HIV. Heterosexual men in sub-Saharan Africa are under-represented in HIV care, yet PCC interventions for men are lacking. We identified core components of a PCC intervention for men living with HIV (MLHIV) in Malawi from both client and healthcare worker (HCW) perspectives, as well as strategies for implementation in routine settings.

**Methods:**

MLHIV≥15 years and not in care were enrolled in parent randomised trials to test the impact of male-tailored HIV services on 6-month treatment outcomes (n=1303). Clients received a PCC package including male-specific counselling+facility ART distribution or outside-facility ART distribution. 50 male clients were recruited for qualitative in-depth interviews using stratified random sampling to assess perceptions of the PCC packages. Focus group discussions were conducted with HCWs who delivered the intervention to understand implementation strategies and potential considerations for scale-up in routine settings. Interviews were audio recorded, translated into English, transcribed and coded in Atlas.ti V.9 and analysed using thematic analysis.

**Results:**

36 MLHIV and 20 HCWs (10 lay cadre and 10 nurses) were interviewed between February and July 2022. Positive interactions with HCWs—characterised by kindness, reciprocity, privacy and focused conversations—and compelling, relevant counselling were considered the most important components of male PCC. While outside-facility ART dispensing was considered helpful, it was not as critical as these other components. HCWs outlined five steps to implementing male PCC: begin with kindness, apologise for past negative interactions, understand men’s holistic story, provide tailored counselling and support development of strategies for adherence. HCWs believed that male PCC enhanced their ability to support male clients but emphasised the need to be integrated into routine services.

**Discussion:**

PCC strategies that foster positive HCW relationships and addresses men’s unique experiences are highly valued by MLHIV. HCWs identified several strategies for delivering PCC to MLHIV that may help close gaps in HIV care for men.

**Trial registration numbers:**

NCT04858243; NCT05137210.

**WHAT IS ALREADY KNOWN ON THIS TOPIC**

- Men in sub-Saharan Africa are particularly prone to cycling through antiretroviral therapy programmes, with more frequent treatment interruptions and extended periods outside of care as compared with women.
- Person-centred care (PCC) leads to improved clinical outcomes for people living with HIV, yet PCC interventions for heterosexual men are lacking.

**WHAT THIS STUDY ADDS**

- When offered a PCC package tailored to men, male clients valued positive interactions and tailored counselling more than community-based ART distribution.
- Interactions were what mattered: how men were treated was considered more important than what service was provided.
- Implementing healthcare workers employed five key strategies for PCC implementation: kindness, apologising for past negative interactions, understanding men’s holistic story, providing tailored counselling and supporting development of strategies for adherence.

**HOW THIS STUDY MIGHT AFFECT RESEARCH, PRACTICE OR POLICY**

- Male-specific PCC can be implemented in routine settings with low-cost training and adequate staff support.
- Equipping healthcare workers to better support men could start to address the persistent gaps in the HIV cascade for men in sub-Saharan Africa.
- The process of implementing tailored care could also positively impact women, adolescents and other subpopulations living with HIV.

## Introduction

 For over a decade, heterosexual men living with HIV (MLHIV) in sub-Saharan Africa (SSA) have had poorer health outcomes than women across the HIV cascade.^1 2^ Men are particularly prone to cycling through antiretroviral therapy (ART) programmes^3^ with more frequent treatment interruptions (TI) and extended periods outside of HIV care as compared with women.^4^,^7^ These differences result in higher rates of advanced HIV disease among MLHIV, which contributes to continued onward transmission and increased rates of mortality.^8 9^

Improving men’s utilisation of HIV services and retention in ART care is essential. It is clear that tailored, responsive interventions can improve health outcomes across a wide range of populations,^10^,^12^ but there are limited interventions specifically for men’s retention in HIV care.^13^,^15^ A critical gap for developing low-cost, scalable interventions is understanding what are essential components of tailored interventions for men. Such understanding can support the development of scalable interventions within SSA, where feasibility and resource considerations are essential.

Person-centred care (PCC) improves use of HIV services and improve clinical outcomes.^16^,^18^ PCC interventions include the following dimensions: (1) addressing barriers to care by increasing convenience and accessibility of services; (2) providing friendly services and (3) increasing responsiveness to client’s needs.^19^ PCC can address some common barriers to care for men, such as inflexible services and long wait times that pose challenges to men’s wage-earning abilities and related travel,^20^,^23^ and fear of unwanted disclosure and anticipated stigma in clinic settings.^24^,^26^ Likewise, skills-based training that sensitises healthcare workers (HCWs) to the unique needs of men^27 28^ and provide tangible communication skills for engaging men^29 30^ can improve outcomes for men, yet such interventions are very limited and often are not feasible within routine settings. To our knowledge, there is no practical guidance for implementation of male-specific PCC interventions that considers feasibility and scalability in routine settings.

We conducted a qualitative substudy within two randomised trials focused on improving treatment outcomes for men experiencing TI in Malawi to identify the most valued components of a short-term PCC intervention for men. We also interviewed implementing HCWs to understand their perceptions of the best strategies for implementing PPC for men and considerations for implementation in routine settings.

## Methods

### Setting

Two randomised control trials (Engaging Men Through HIV Self-Testing with Differentiated Care to Improve ART Initiation and Viral Suppression in Malawi (ENGAGE)^31^ and Identifying Efficient Linkage Strategies for Men (IDEaL))^32^ tested the impact of male-tailored HIV services on men’s 6-month treatment outcomes in Malawi. Malawi’s adult HIV prevalence rate is 7%; there are approximately 360 000 MLHIV in the country, of whom nearly 52 500 (15%) are not currently on ART.^33^

MLHIV who were ≥15 years, living in study facility catchment areas, and not currently in HIV care (defined as never initiated ART or experiencing TI of >28 days) were eligible for enrolment in the parent trials. Within the parent trials, study teams reviewed routine medical charts and health registers to identify eligible men at 24 participating health facilities across the central and southern regions. Men were then traced in the community, screened, and if eligible, provided informed consent and randomised to either standard of care or one of the three intervention arms described below:

- Male counselling arm: One-time male-specific counselling+facility navigation and facility based (re)initiation.
- Home-based ART (1 month): One-time male-specific counselling+immediate outside-facility ART initiation (1-month dispensing)+facility navigation for follow-up ART visits.
- Home-based ART (3 months): Male-specific counselling+outside-facility ART initiation for 3 months+facility navigation for follow-up ART visits.
- Staged arm: Intervention activities build in intensity over time for men who do not reengage in care within 2 weeks: (1) one-time male-specific counselling+facility navigation; (2) male mentorship+facility navigation and (3) male-specific counselling+outside-facility ART initiation (1-month dispensing)+facility navigation for follow-up ART visits.

A total of 1303 MLHIV were enrolled across the two trials (734 ENGAGE, 569 in IDEaL).

### Male-specific PCC package

All enrolled clients received male-specific counselling based on the standard HIV testing and counselling curriculum from the Malawi Ministry of Health. Methods for developing and piloting the male-specific counselling curriculum developed for the parent trials are described in detail elsewhere.^34^ In brief, the curriculum was developed through an iterative co-creation process involving HCWs, MLHIV and other stakeholders to ensure feasibility and contextual relevance. Routine, gender non-specific standard of care counselling content was modified using existing literature and formative qualitative research^2035^,^39^ to specifically focus on men’s concerns related to ART adherence and barriers to care. We also developed a supplemental visual aid featuring motivational depictions of men living successfully with HIV for use in counselling sessions (online supplemental information 1).

Two HCW cadres carried out all PCC activities: a lay cadre of community HCWs and nurses. All HCWs who implemented the interventions were male. Lay cadre had a minimum qualification of a high school diploma and were responsible for HIV-related tracing activities and counselling in health facilities. Nurses had a degree in nursing, had previous experience in HIV counselling and were responsible for delivering HIV counselling and treatment services both in facility and community settings. All HCWs received a 2.5-day training conducted by study facilitators trained on male-specific PCC. Topics included in the PCC training were: HCWs sensitisation to the challenges and needs of men as ART clients (0.5 days); best practices for implementing PCC (0.5 days); review of curriculum (0.5 days) and peer role play and feedback on counselling strategies (1 day).

Male-specific counselling and outside-facility ART dispensing were provided in the community in locations of the participant’s choice (home or elsewhere). Counselling was conducted in the local language (Chichewa) and lasted 40–70 min. Clients were asked if they would like to reinitiate ART following the first counselling session and if they chose to do so, were provided either outside-facility ART dispensing or an escort to the facility for dispensing depending on the assigned study arm. If returning to the facility, clients were provided with navigation to familiarise them with facility processes. HCWs were provided with airtime for tracing clients but were not required to maintain contact with participants outside of protocol-driven time points.

## Qualitative methods

### Participant in-depth interviews

We used random sampling to identify a subset of study clients for qualitative in-depth interviews (IDIs), stratified by intervention (male counselling only, male counselling+home-based ART 1 month or 3 months). Clients were eligible if they were enrolled in the parent trials within the past two to three months and randomised via the parent trials in either of the aforementioned arms. All interviews were conducted two to three months after study enrolment to limit recall bias. Actual exposure and level of interaction with interventions were not part of eligibility or selection criteria.

An English interview guide was developed to elicit information on the following domains: (1) pretrial experiences with ART; (2) perceptions of the male-specific PCC package; (3) experiences with study HCWs and (4) motivations for ART reinitiation (or not). The guide was then translated into the local language (Chichewa) and piloted with seven clients. The guide was revised and further refined based on feedback by the implementing research assistant, research coordinator and translators (online supplemental information 2). Selected clients were traced via phone if a phone number was provided at the time of parent enrolment, or through community tracing based on information provided by the health facility and confirmed by implementing HCWs. Interviews were conducted in Chichewa by a trained male research assistant in private locations within the community. Interviews lasted 40–120 min and clients were provided with local currency equivalent to US$10 for their participation in line with local ethical guidelines.

Interviews were audio recorded, translated into English, transcribed for analysis and reviewed for quality by a third party. An initial codebook was developed based on the literature on facilitators and barriers to men’s HIV care engagement used for developing the male PCC curriculum as well as the interview guide. The codebook was piloted on eight transcripts by two investigators and inductive codes were added where necessary. Transcripts were then coded in Atlas.ti V.9^40^ using inductive and deductive methods and were independently reviewed to ensure consistency. Analysis was then conducted using thematic analysis centering on clients’ experiences with the PCC curriculum and implementing HCWs, with particular focus on identifying similarities or differences between study arms (male counselling only, male counselling+home-based ART 1 month or 3 months) and reasons for ART engagement.

#### HCWs focus group discussions

We conducted focus group discussions (FGDs) with implementing HCWs to explore strategies for implementing male-specific PCC services and to identify potential implementation challenges in routine settings. Interview guides were used to encourage HCWS to reflect on the PCC counselling curriculum and assess barriers and facilitators to implementing the curriculum with fidelity. The guides also included prompts for self-reflection to elicit personal experiences as well as longitudinal perceptions of curriculum (online supplemental information 3). All HCWS were brought together in person for FGDs as a part of ongoing study-related trainings held in Lilongwe, Malawi’s capital and provided informed oral consent for FGD participation. FGDs were conducted separately for lay cadres and nurses by trained male facilitators in both Chichewa and English based on participants’ preferences. Four FGDs were conducted in total: two at 6 months and two at 9 months after study activities began. FGDs lasted 90–150 min. Discussions were audio recorded, translated into English where needed and transcribed and reviewed for quality by a third party. Data were coded by two investigators using inductive and deductive methods in Atlas.ti V.9^40^ and analysed using thematic analysis.

### Patient and public involvement

This substudy was a part of the IDEaL and ENGAGE randomised control trials. Formative qualitative research was conducted with MLHIV in local communities to develop the PCC package, and both MLHIV and local HCWs participated in qualitative research to further refine the PCC package prior to implementation. Dissemination to participating male clients, HCWS and participating health facilities will be conducted the following the completion of the parent trials. All data collected and used for the writing of this article is publicly available.^41^

## Results

### Participant demographics

A total of 50 male clients were selected for IDIs. 36 men were successfully contacted and interviewed between February and June 2022. Men had a median age of 36 years, were predominantly married (n=23) and one-quarter had completed secondary school (n=9). The majority had received male counselling only (n=15) or male counselling and home-based ART dispensing for 1 month (n=15). Half of the clients had been on ART for 1 year and had a mean of 122 days outside of care prior to the intervention. All but two had reinitiated ART at the time of interviews.

20 male HCWs (10 lay cadres and 10 nurses) participated in FGDs in April and July 2022. HCWs had a median age of 31 years. The majority had been providing HIV services for two or more years prior to the study (table 1).

**Table 1:** Characteristics of male clients (n=36) and HCWs (n=20) at study entry

Male client characteristic	Total
	(n=36)
Demographic variables	
Median age, (IQR)	36 (28–43)
Married, n (%)	23 (64)
Attended any secondary school, n (%)	9 (25)
Mean number of children with current partner, (IQR)	1 (0–2)
Trial and arm, n (%)	
IDEaL: Male-counselling	15 (42)
IDEaL: Male-counselling+home-based ART (1 month)	15 (42)
ENGAGE: Male-counselling+home-based ART (3 months)	6 (16)
District, n (%)	
Central	24 (67)
South	12 (33)
ART services	
Median years on ART, (IQR)	1 (0.5–4)
Mean days out of care at enrollment, (IQR)	122 (38–199)
**HCW characteristic**	(n=20)
Cadre	
Lay cadre	10 (50)
Nurse	10 (50)
Demographic variables	
Mean age, years (IQR)	31 (27–35)
Mean years of experience in HIV services, years	2

ARTantiretroviral therapyENGAGEEngaging Men Through HIV Self-Testing with Differentiated Care to Improve ART Initiation and Viral Suppression in MalawiHCWshealthcare workersIDEaLIdentifying Efficient Linkage Strategies for Men

### Client perspectives

**Figure 1:**
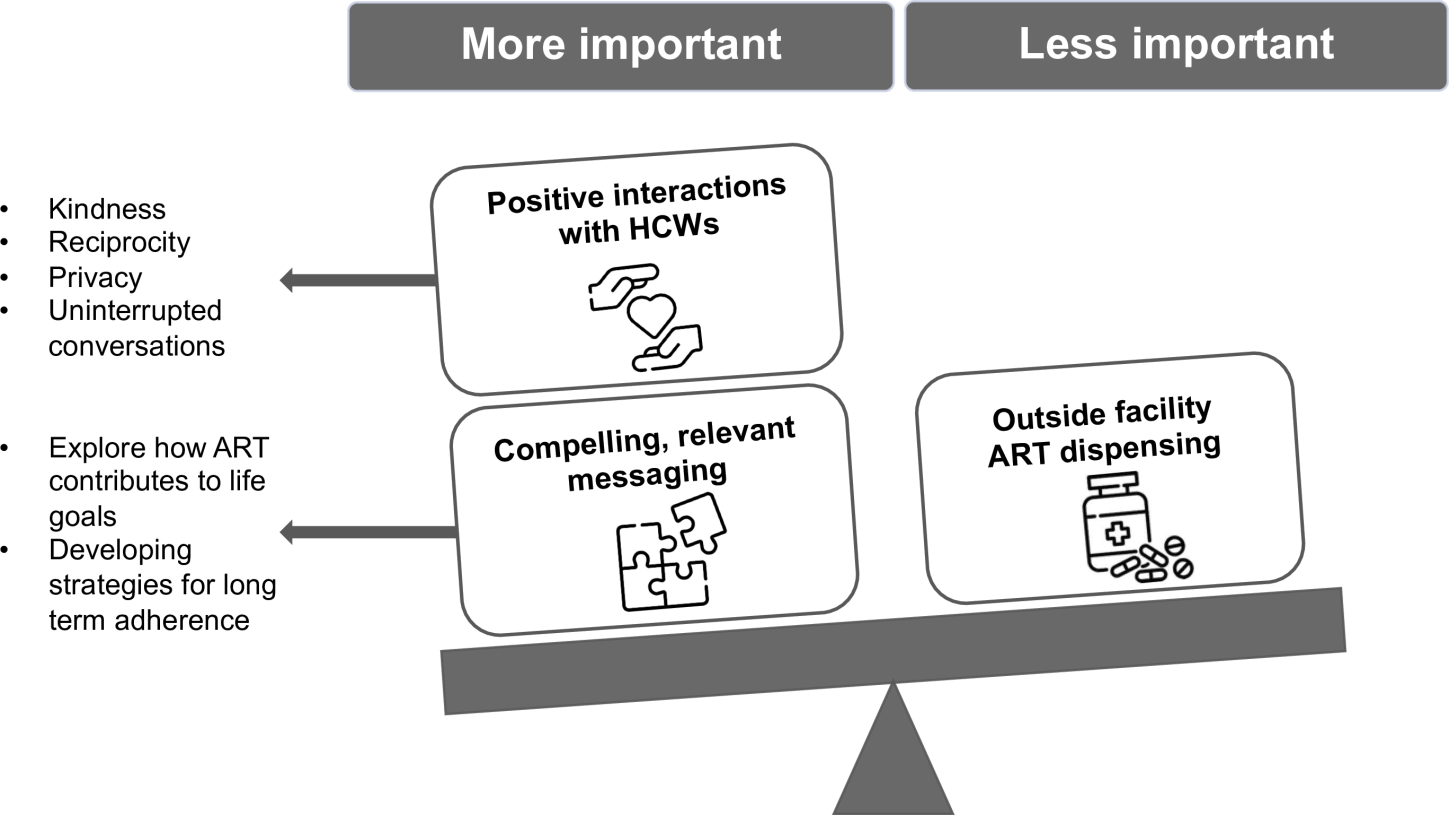
Male client preferences for male-specific PCC. Scale graphic. ART, antiretroviral therapy; HCWs, healthcare workers; PCC, person-centred care.

Clients identified two core components of male PCC: positive HCW interactions and compelling, relevant messaging (figure 1).

#### Outside-facility dispensing

Among clients who received outside-facility ART dispensing as part of the intervention (n=21), only three said they (re)initiated ART due to this service. While ART dispensing outside the facility was described favourably, almost all considered the other two factors to be more influential than outside-facility ART dispensing (figure 1).

It [outside-facility dispensing of ART] was an easy way to return to care. But I liked the counselling that he gave me most. I felt well informed on what to do and how to do it [engage in ART programs]. We discussed my [adherence] challenges and how to avoid them next time. I went [back to the facility] without fears afterwards. – *Male client, 60–70 years*

#### Positive interactions with HCWs

Clients were strongly motivated by the positive HCW interactions male-PCC provided. 34 (94%) clients reinitiated ART after the PCC intervention. The vast majority attributed their decision to re-engage to positive interactions with study HCWs, while a minority reengaged due to poor health or change in circumstances (eg, returning home following work-related travel). Prior to the intervention, negative experiences with HCWs were common. One-third (n=13) of clients said such experiences were the primary reason for stopping ARVs or failing to re-engage in treatment. Following the intervention, 33 clients (91%) reported having an improved relationship with study HCWs.

Four fundamental aspects of positive HCW interactions emerged: kindness, reciprocity, privacy and uninterrupted and focused conversations.

First, clients positive interactions began with feeling treated like family. Showing respect, kindness and interest in men’s welfare beyond just providing ART services facilitated familial interactions, while HCWs’ willingness to share personal information and engage in reciprocal conversations deepened feelings of closeness. Clients most commonly reported a closer bond to lay cadres than nurses; however, clients felt that their experiences with both cadres were far better than previous experiences with HCWs.

The [study] HCW encourages me the same way a person would encourage their relative. – *Male client, 20–30 years*

Their behavior is that of love and caring towards my life. – *Male client, 30–40 years*

For the majority of clients, developing a close bond was dependent on reciprocity, relating to one another as fellow men and discussing topics that men cared about outside of their HIV status, such as income generation, family, sports and relationships.

He opened up to me as a fellow man and there was nothing to hide. So, I also opened up to him. – *Male client, 30–40 years*

The way we interacted was so open, he was open about everything he said and he was never angry. – *Male client, 30–40 years*

Clients particularly valued when HCWs understood men’s concerns about privacy and took active steps to avoid stigmatising situations during interactions in the community. Fear of stigma was a prominent concern for nearly half of the clients (n=15). Ensuring privacy helped clients build trust with HCWs which enabled them to then be more open.

They [HCWs] keep our secrets. I trust them because we chat in secluded areas to make sure that what we discuss will remain private. – *Male client, 20–30 years*

When [the study HCW] visits me [in the community] he is incognito. He doesn’t reveal why he has come to my house. – *Male client, 30–40 years*

During counselling, clients noted that HCWs were also able to provide their undivided time and attention unlike in routine healthcare settings. Clients did not feel rushed or interrupted, allowing for more time to ask questions and overall higher quality interactions.

He is direct and empathetic to me, whilst the other HCWs would just leave you and attend to other things in the course of the counselling session. – *Male client, 30–40 years*

[Past HCWs] spent little time with us and their topics were cut short. But these HCWs take time to ask what challenges I’m facing now, and what I faced in the past. – *Male client, 20–30 years*

#### Compelling, relevant messaging

The second core component identified was compelling, relevant messaging. Tailored counselling that focused on men’s specific goals in life and roles in their family, rather than simply sharing standard health information, was considered more compelling than the standard of care. Tailored counselling signalled to men that HCWs were truly focused and interested in them as individuals. More than one-third of clients described learning something new about the connection between ART and their individual goals and hopes for the future.

I learned that by taking the treatment regularly, one can be strong and take care of his family. This was the new understanding for me, different from the previous counselling that focused on ARVs prolonging life for an individual only. – *Male client, 30–40 years*

I learned that if I adhere to ARVs, I can have good health and carry on with work just like anybody else who is HIV negative. This counselling was different. It was like I was seeing things on the ground [relevant to daily life]. – *Male client, 40–50 years*

An aspect of compelling counselling deeply valued by clients was the ability to develop strategies for staying in care with HCWs. Clients felt a sense of empowerment when HCWs worked with them to identify previous and future potential barriers to care and develop tangible strategies to overcome them. One-third (n=12) of participants outlined specific strategies they had developed with the support of their HCWs (table 2).

**Table 2:** Strategies developed to overcome future challenges to ART adherence

Barrier to care	Solution strategy	Quote
Missed pills due to travel	Multimonth dispensing, transfer letter, emergency refill	Now when I’m going to work away from home, I collect some of the ARVs and put them in a plastic bag and have them with me. That way I avoid skipping when I travel. – *Male client, 30–40 years*
Lack of support	Status disclosure	People were not aware of my HIV status, so when I would miss work for my refill, my employer would wonder. The HCW encouraged me to open up to my boss so that he could understand the situation. I used to be shy about discussing this, but now that I explained everything to my boss. Now he has become someone who encourages me to go and collect ARVs. – *Male client, 30–40 years*
Difficulty with lifelong medication	Reframing adherence requirements	I told him taking medication everyday was a burden for me. We agreed I should think of taking treatment like eating nsima [maize staple food], which I never get tired of. Actually, taking ARVs is even simpler because its only once a day unlike nsima which is eaten twice a day! – *Male client, 50–60 years*
ART and alcohol	Taking medication despite drinking	When I got drunk, I would decide not to take ARVs. But the HCW said that one can do both. It’s not like you take alcohol the whole day. You can take ARVs in the morning then go for a drink at night. This counseling helped me. – *Male client, 30–40 years*

ARTantiretroviral therapy

### HCWs’ strategies for implementing PCC

**Figure 2:**
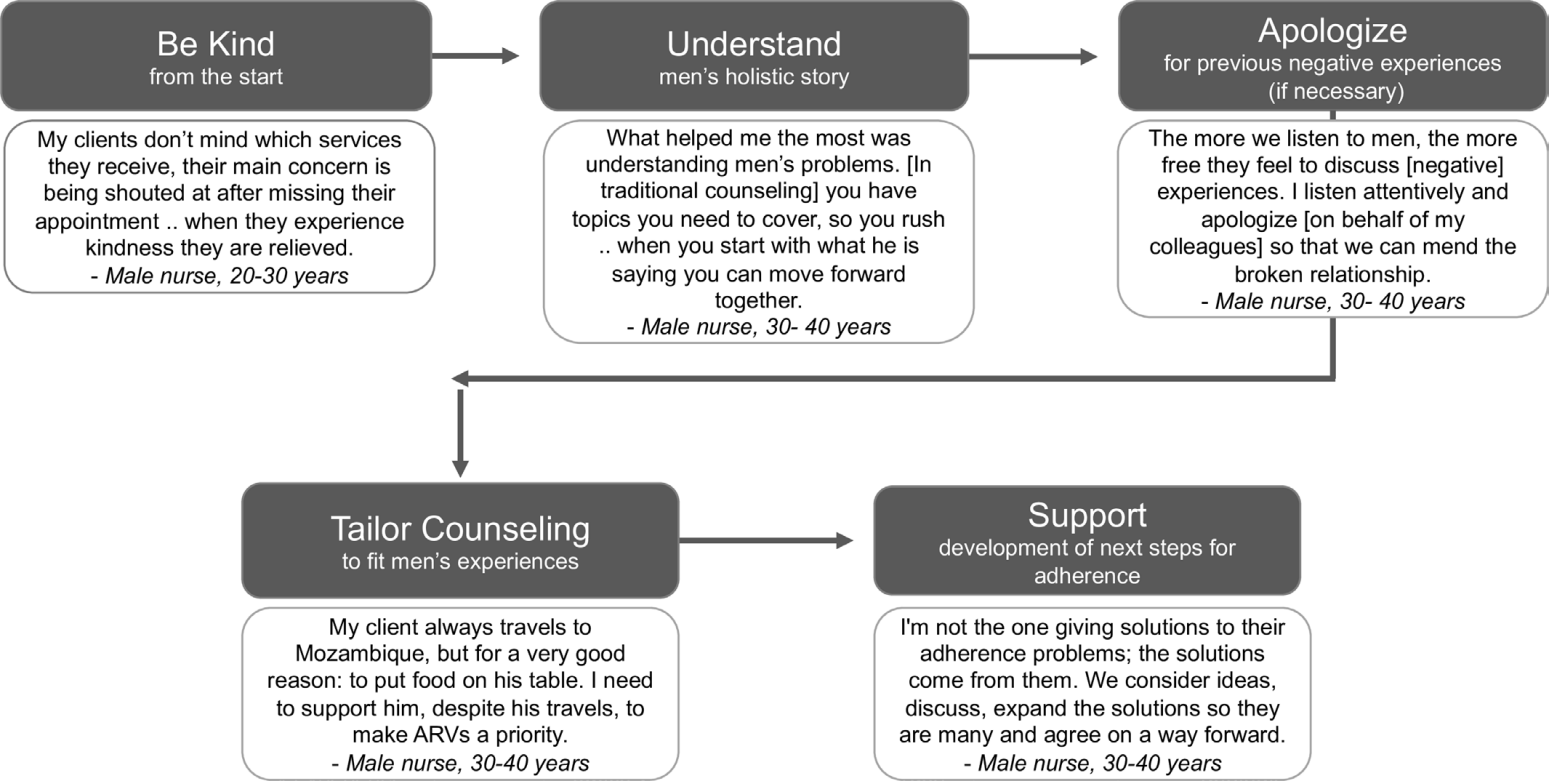
Summary of strategic steps for implementing male-PCC. Flow chart graphic. PCC, person-centred care.

When asked for their strategies for implementing male-specific PCC, HCWs described five key steps (figure 2 and online supplemental information 4). Findings were similar across nurses and lay cadre.

HCWs agreed that treating men with kindness and respect from the first interaction was essential. Many men anticipated negative interactions with HCWs based on their past experiences. HCWs reported that when approached with kindness, male clients spoke more frankly about their struggles with adherence because they did not fear judgement or harsh treatment. For those with particularly bad previous experiences, HCWs believed it was helpful to apologise on behalf of the health system to ‘mend broken relationships.’ Apologising was thought to be an important step to help male clients feel that their experiences were acknowledged by the health system to regain their trust.

HCWs then sought to understand a male client’s life holistically: where he lived, the nature of his work, his relationships with others, his demands at home and hopes for the future. To be effective, HCWs needed to listen actively. HCWs could then provide relevant, compelling counselling by connecting the potential impact of sustained ART adherence with valued parts of men’s lives.

HCWs acknowledged that understanding men’s reasons for defaulting was only the first step to supporting men’s use of HIV services. HCWs needed to foster clients’ problem-solving skills and support clients development of their own strategies to overcome future challenges. Actively encouraging and supporting self-developed strategies for success allowed HCWs to (1) affirm that lifelong medication can be challenging and (2) help clients develop a sense of self-compassion and readiness for future adherence challenges.

HCWs described how PCC strategies also improved their own experiences. PCC increased HCWs’ sense of confidence and job satisfaction as they felt they were able to provide practical guidance that could make a difference. Learning about clients’ unique challenges helped HCWs understand the multiple barriers to men’s adherence and increased their own empathy towards men as ART clients.

#### Strategies for scale up and sustained PCC

HCWs proposed several strategies to take PCC to scale. First, general HCWs could be sensitised to the benefits of PCC, especially the positive impact that implementing PCC strategies can have on HCWs themselves. HCWs in our study believed that using male PCC increases HCWs sense of confidence and job satisfaction as they felt better equipped to support clients and make a tangible difference.

[Through male-specific PCC training] we have sharpened our skills. There were male clients that HCWs said would never return back to care, but because of the [PCC] training and expertise, we were able to use a different approach and the results show [client returned to care]. This has boosted my confidence a lot. *– Male lay cadre, 30–40 years*

Most HCWs emphasised that PCC could optimise HCW time and effort. For example, HCWs discussed how their colleagues could avoid spending unnecessary time following a counselling script that includes information that is either already known or not relevant to clients by asking clients about their challenges and tailoring counselling to the most relevant challenges facing that client. This was particularly relevant for clients experiencing TI who often had been taking ART for some time and did not require general information, but responsive support.

Counselling is not only a one-sided thing counseling, it requires getting each other involved. You can finish the whole [standard counselling] and clients will listen but they will not tell you their problems. It is better to be open, ask them questions and allowing them to also ask you questions, and in so doing you are able to discuss things that you didn't know or wouldn't have known if you had just used [standard counselling]. – *Male nurse, 30–40 years*

HCWs also recommended that male-specific PCC not be siloed as a separate intervention. HCWs believed that PCC approaches should be embedded within routine facility procedures during any ART visit. HCWs agreed that PCC integration should include training, monitoring and evaluation to support best practices, and accountability for HCWS who mistreat male clients.

Men default because they feel that they are not spoken to with respect. We should be targeting the culprits of such things, educating them about respect and understanding the situation of clients. We are scaring men away, and when they default, we have to start over again. – *Lay cadre, 30–40 years*

## Discussion

In this qualitative study, we sought to identify the most valued components of a short-term PCC intervention for men experiencing TI in Malawi, and HCW perceptions of the best strategies to implement and scale up PCC for men. We found that male clients prioritised positive and personable interactions with HCWs, characterised by kindness, reciprocity, privacy and uninterrupted conversations, as well as motivating counselling messages that were tailored to men’s specific needs and priorities. Importantly, men valued positive interactions and tailored counselling more than community-based ART distribution. HCWs also believed that kind, positive interactions were the most important strategy to improve men’s engagement. They identified five simple steps to foster these interactions: show kindness, listen and understand men’s experiences, acknowledge past negative experiences with HCWs and health services, provide tailored responses and support men to make a plan for staying in care in the future.

Our findings provide important insights into men’s preferences regarding HIV services and the critical importance of positive relationships with HCWs. While outside-facility ART dispensing was appreciated, it was not considered as essential as positive interactions with HCWs. It is established that poor interactions with HCWs negatively impact ART engagement among both men and women.^42^,^46^ HCWs often perceive men as ‘selfish’ ‘prideful’, ‘overly self-reliant’ and ‘difficult clients beyond reach’.^36 47 48^ These biases can impact HCWs approach to and motivations for providing men with quality services, particularly when coupled with frustrations towards those who experience TI.^42 43 49 50^ Men have few opportunities to interact with health services^51^ and have much less experience navigating health systems compared with women.^36 39 52^ Consequently, negative interaction with HCWs may have large implications for how they choose to engage with health systems in the future. Other literature on chronic care emphasises the importance of positive interactions with HCWs,^45 53^ client satisfaction and client empowerment for improved health outcomes.^54^,^56^ Structural changes such as differentiated service delivery models that reduce wait times,^57^ reduce facility visits,^58^ provide ‘male-friendly’ services^59 60^ and community outreach^61^,^64^ can effectively reach men and are urgently needed. Yet these interventions on their own are not enough. PCC programmes for men should integrate a focus on improving men’s experiences within the health system alongside interventions to improve men’s access to services themselves.

Male-specific PCC benefited men and HCWs alike. Participants connected best with compelling counselling messaging that spoke directly to their individual priorities and concerns. They particularly valued understanding how ART adherence could support their future goals, supporting findings within the region that focusing on the positive benefits of ART, or ‘flipping the script’, improves men’s motivation and engagement in care.^65 66^ For HCWs, using PCC increased satisfaction and pride in their work. HCWS are often ill equipped to address issues of importance for men.^37 48^ Study HCWs expressed improvements in their ability to understand the challenges men faced through active listening and felt engaged with clients in the co-creation of strategies for long-term adherence. Implementing PCC strategies can create positive feedback loops for HCWs: having kind, engaged interactions can increase HCWs ability to understand and relate to clients, increasing HCW empathy and job satisfaction knowing that they are making a meaningful difference.^67^ This positive feedback can help to reduce HCW burn-out, a persistent challenge in the region^68 69^ and improve clients’ own agency and empowerment.^70 71^

Our findings may also have implications for other ART client groups. All clients can benefit from PCC.^17^ The process of developing tailored curriculum and the five steps described by HCWS for implementing PCC could also positively impact women, adolescents and other priority populations. Future studies may consider how PCC materials from our study may translate to other subpopulations.

How can PCC be implemented into routine services? Our findings suggest it is possible. We found that when it comes to PCC for men, interactions were what mattered: how men were treated was considered more important than what service was provided. First, treating clients with kindness and having reciprocal conversations is possible within existing health systems. Even when staff and resources are limited, quality of interactions with HCWs can be more impactful than quantity of time spent with clients.^53 72^ Focusing on kindness does not require a complete overhaul of the health system, but can be taught and have a big impact.^45 73 74^ Second, HCWs are able to implement PCC techniques when adequately supported. When HCWs are equipped with the skills and support needed to delivery high-quality care, they can make the most of their limited time with clients.^75^ Low-cost tools such as job aids with open-ended questions and prompts for non-judgemental language embedded in counselling curriculum can help make routine services more client-centred.^45^ Active supervisory feedback and knowledge sharing among HCWs can further support PCC interactions within clinics. Finally, male clients believed lay cadres were better positioned than nurses to implement meaningful PCC strategies. Peer support models are a cost-effective way to improve initiation and retention outcomes for men^76 77^ and should be further explored.

Our findings should be interpreted in light of the following limitations. First, we draw from a small sample (n=36) of men who may not represent all men or capture the nuance of HCW interactions men experience. Quantitative analysis of HCW interactions within the IDEaL and ENGAGE trials is in process. Second, the 3-month home-based dispensing arm was under-represented in our sample (6 participants compared with 15 in the other arms). However, in subanalysis, we did not identify any differences in themes between the 1-month and 3-month home-based arms. Last, HCWs participated in FGDs, which may have introduced social desirability bias or a reluctance to share opposing viewpoints against the PCC intervention in a group setting.

### Conclusion

When we bring men to the centre of HIV care and listen to their needs, we find that they want kind, tailored and empowering services. PCC strategies that foster positive HCW relationships and tailored counselling that address men’s unique experiences are desired and prioritised by MLHIV. HCWs identified several strategies for delivering PCC to MLHIV that may help bridge the gaps in HIV care for men. Equipping HCWs to better support men could start to address the persistent gaps in the HIV cascade for men in SSA.

## supplementary material

10.1136/bmjph-2024-001100 online supplemental file 1

10.1136/bmjph-2024-001100 online supplemental file 2

10.1136/bmjph-2024-001100 online supplemental file 3

10.1136/bmjph-2024-001100 online supplemental file 4

## Data Availability

Data are available in a public, open-access repository.
