# Supplementary material for: Core components of male-specific person-centred HIV care: a qualitative analysis from client and healthcare worker perspectives in Malawi
Source: BMJ Public Health. 2024 Dec 22;2(2):e001100. doi: 10.1136/bmjph-2024-001100 (PMC11816952; doi:10.1136/bmjph-2024-001100)
Supplement: online supplemental file 1 [file bmjph-2-2-s001.pdf]

Supplemental Information 1: Examples of male-specific messaging images

Male-specific messaging	Picture
ARVS work as a shield and protect your body from infections.	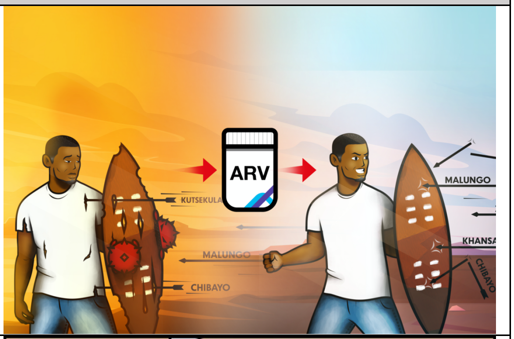
Importance of and strategies for long term adhere	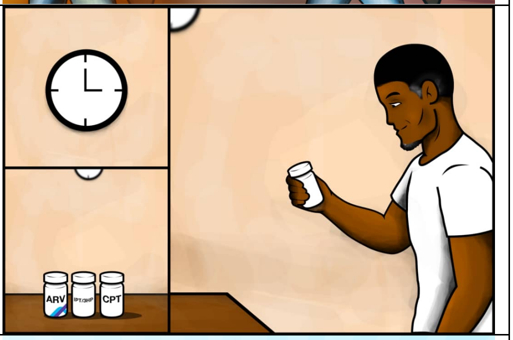
Protecting sexual partners through treatment and prevention and condoms	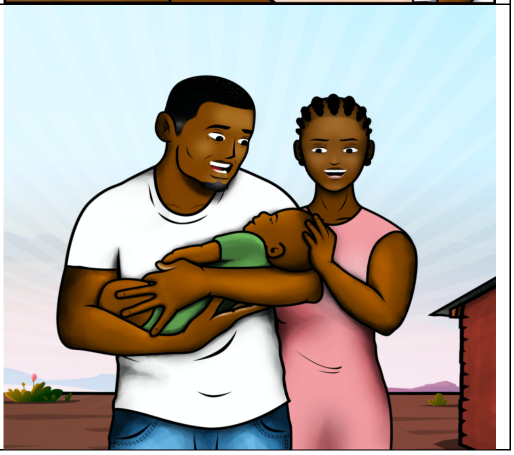
