# Supplementary material for: Core components of male-specific person-centred HIV care: a qualitative analysis from client and healthcare worker perspectives in Malawi
Source: BMJ Public Health. 2024 Dec 22;2(2):e001100. doi: 10.1136/bmjph-2024-001100 (PMC11816952; doi:10.1136/bmjph-2024-001100)
Supplement: online supplemental file 2 [file bmjph-2-2-s002.pdf]

Supplemental Information 2: In-Depth Interview Guide for Male Clients

---

Pre-Trial Experiences
To start, I'd like to briefly talk about your experience with HIV services before you were enrolled in the study.

1. At some point in the past, you stopped taking ARV's (or didn't start ART), which is why you were recruited into this study. Can you walk me through some of the reasons that it was difficult to stay on (or start) ART in the past?

Personal	Male Role	Friends/Community	Health Facility
• *Concern about ART side effects*<br>• *Concern about remembering to take medication every day*<br>• *Concern about having to be on ART for the rest of their lives*<br>• *Why take ART when healthy?*	• *Travel associated*<br>• *Work/earning money has to stop in order to visit facilities*<br>• *Unable to take off work to for appointments*	• *Inability to disclose to partner*<br>• *Fear of disclosure to others (family, friends, community)*<br>• *Religion*	• *Lack of privacy in the facility*<br>• *Distance to the facility*<br>• *Wait times at the facility*<br>• *Negative experience with health care workers*

Trial Experience
As you know, the reason I am speaking with you now is because you were enrolled in a study, and now I'd like to understand your experience with the study. I'd like to understand your experiences AFTER you were enrolled in the study. Please think about the time since you were first given 8,000 MKW until now.

1. As a part of your participation in this study, you may have been visited a couple of times by different people. Did anything **bad** happen as a result of these visits? (i.e. the visits when you were given 8,000 or the visit(s) by a health care worker (Patient Supporter/Nurse)?

2. Did someone discover your HIV status as a result of the study or as a result of taking ART since you enrolled (since the first time you got the 8,000)?
    1. *If yes:* What happened exactly? Who discovered your status, and how?

3. Since you received 8,000 for the first time, how many times have you met with a health care worker (Patient Supporter/Nurse) about HIV (<u>excluding ART clinic visits</u>)?
    1. *If yes:* Where did you meet this person? Probe: at your home, at your work place, in the community (church, school, someone elses house), near the facility?

4. When this health care workers (Patient Supporter/Nurse) met with you, you may have gone through this Men's Counseling Flip Chart [SHOW THE FLIP CHART].
    1. Have you ever seen this flip chart? *If NO, move to question 4.2. If Yes, continue.*
        i. IF YES: Can you describe briefly what you discussed when the health care worker/patient supporter used this flip chart?
        ii. IF YES: How was the counseling from this flip chart different from the counseling you have received in the past (before joining the study)?
            1. Were there information covered that you didn't know before?

2. IF NO: Was the information you already knew presented in a new or different way in the flip chart?
3. Did you feel like the way the health care worker/patient supporter spoke to you was different then previous health care workers? (exp: was talking directly to you -tailored to you_instead of being very generic?)
4. Did you enjoy the pictures/art? If so, what did you like about them?
4. Did the health care worker/patient supporter ask you more questions then previous HCWs?

iii. IF YES: What topic/idea from the flip chart was the most helpful to you? (NOTE: let them flip through the Flip Chart)

iv. IF YES: You described to me earlier that you have missed ART in the past because of [X] reason. Did the new counseling with this flip chart help you deal with [X]? How?

1. Did the counselor discuss a plan with you about how to overcome [X] or any other challenge that made taking your medication regularly difficult?
   a. If YES: What was the plan about?
   b. If NO: Do you wish they would have help you more on this?
2. Are there any topics or questions about HIV/ART that you you wish were covered or covered more during counseling?
   a. IF YES: What are they?

2. IF NO: When you met with the health care worker, can you briefly describe what you discussed?

i. Were there new topics or ideas you discussed that you didn't know before?

ii. Was the information you already from the flip chart knew presented in a new or different way?

iii. Did you feel like the way the health care worker (Patient Supporter/Nurse) spoke to you was different then previous health care workers? (exp: was talking directly to you -tailored to you instead of being very generic?)

iv. Did the health care worker/patient supporter ask you more questions than previous HCWs?

5. As a part of the study you may have been offered to be given ART in the home/community. Did you decide to get ART at home/in the community? *If NO: Skip to Question 6.*

a) IF YES: Please describe what happened the day you were given ART at home.

i. Did the one who gave you ART at home counsel you? What was the most helpful thing he said?

ii. Do you have any additional questions you think still need answered?
IF YES: What is it?

6. How do you currently get ARTs?
   1. IF at HOME/COMMUNITY: How many times have you gotten your medication at home? How many pills were you given (i.e. 30 day supply, 90 day supply?) Is home-based dispensing still happening?
   2. If at the FACILITY: When you went back to the facility, did the did a health care worker/patient supporter offer you any other services?

i. What did they do?

ii. Was this helpful for you? Why/why not?

7. Has either HCW been supporting you or chatting with you about anything else in addition to HIV or ART?
   1. Do you ever contact either HCW directly to chat or ask questions?

i. IF YES: How often do you talk with them? What do you talk about?

2. Is your relationship with either HCW different to the ones you have had with other HCWs before joing the study? How?

i. IF YES: Do you think this relationship helps you more than your relationship with other HCWs? IF YES: How?

8. Now think about overall your interactions with the health care workers you interacted with since you started the study – the Patient Supporter and the Nurse who brought you ART. What did you like about your interactions with them? How do you think it helps you with ART?
    1. Was there something about their behavior/personality? Or what he said? Or did he help you feel more comfortable at the facility?
    2. Did you like one more than the other? Why?

9. With everything in life, there are some things we like and some things that could be a little better. What did you NOT like about your interactions with the Patient Supporter and the Nurse who brought you ART.? Does this make it more difficult/discourage you to be on ART?
    1. Probe: Was there something about their behavior/personality? Or what they said? Or how you were treated at the facility?
    2. Did you dislike one more than the other? Why?

10. Is there anything else you think you need a health care worker or the health facility could do differently in order for you to be comfortable taking ART regularly? Is there anything else you need?

Initiation
Now I'd like to ask you about what has happened with your ARVs since you were enrolled in the study.

11. Did you start taking ART (again) since enrolling in the study?
    If YES:
    a. Why did you (re) initiate ART? What convinced you it was good to take medication (again)?
    b. Who was most influential in your decision to start ART (again)?
    c. *If they did not mention a study team member:* Of the people we discussed, including the person who [gave you 8,000 MK], who would you say was the most influential in your decision to start ART (again)? What did they do specifically that influence your decision?
    If NO:
    d. Why have you not initiated ART since enrolling in the study (since you got the first 8000MK)?
    e. Has anyone influenced you to not start ART? Who? Why?
    f. Is there anything that could motivate/help you to initiate ART?
        i. What about other men like you who may struggle to initiate ART, what other things (besides the visits you had with the health care workers) do you think *would help them start ART?*
12. We know that starting ART can be difficult. What do you think is the most difficult thing about re-starting ART for you? (potential probes below)

Personal	Male Role	Friends/Community	Health Facility
• *Concern about ART side effects*<br>• *Concern about remembering to take medication every day*	• *Travel associated*<br>• *Work/earning money has to stop in order to visit facilities*	• *Inability to disclose to partner*<br>• *Fear of disclosure to others (family, friends, community)*<br>• *Religion*	• *Lack of privacy in the facility*<br>• *Distance to the facility*<br>• *Wait times at the facility*

• *Concern about having to be on ART for the rest of their lives*<br>• *Why take ART when healthy?*	• *Unable to take off work to for appointments*		• *Negative experience with health care workers*

13. Was there anything that made it easy for you to (re)start ART? (opposite of probes below)
    1. For those who opted for HOME-BASED: Do you think getting ART at home helped you re-engage in care? Why/why not?

14. For the next set of questions please only think about the time since you were given the first 8,000 MKW, not before. Please feel free to be honest. There is no right or wrong answer.
    1. Do you feel MORE confident you can stay on ARTs in the future? Why/why not?
    2. Is there someone in your life now that encourages you?
    3. Do you have a better relationship with HCWs / or trust them more? Why/why not?
    4. Do you have a plan so that you don't run out of ARVs if youre away or busy?
    5. Do you think ARVs help you reach your goals for making money and for your family? Why/why not?

Conclusion

15. Apart from what you've already talked about, is there anything else that you want to share?

16. I am not a healthcare worker, but are there any questions you have for me?
