# Supplementary material for: Core components of male-specific person-centred HIV care: a qualitative analysis from client and healthcare worker perspectives in Malawi
Source: BMJ Public Health. 2024 Dec 22;2(2):e001100. doi: 10.1136/bmjph-2024-001100 (PMC11816952; doi:10.1136/bmjph-2024-001100)
Supplement: online supplemental file 3 [file bmjph-2-2-s003.pdf]

Supplemental Information 3: Focus Group Discussion Guide for Health Care Workers

---

Good morning, we want to have this discussion to understand your experience with providing the IDEaL/Engage interventions. This is not an exam, we believe your thoughts would help us fully understand the flipchart and how we can make it better based on your thoughts. We know the interventions aren't perfect, and that things change when you are in the field. Each of you now have months of experience using it with a range of different men, each with their unique challenges and issues. We want to hear feedback from your experiences with the materials, both positive and negative. There is no right or wrong answer. Feel free to share your experiences and be open.

GENERAL EXPERIENCE

1. What are your general experiences with the IDEaL/Engage Study?
    a. If you had to describe to someone what you have been doing, what would you tell them?

2. What part of your job/responsibilities as a part of the IDEaL/ENGAGE study do you feel most confident in? (Answer with very confident, confident, somewhat confident, not confident) Reminder: This is NOT a performance review, this is so we can understand how you <u>feel</u> about counselling and providing treatment. It will help us to know how to support you in the future, not how to assess your performance. *NOTE: This is about their roles in the study specifically, not just that they are a nurse.*
    a. How confident are you in the male-specific counselling? *(NOTE: Actually providing counselling, not just using the flipchart)*
    b. How confident are you in providing facility navigation?
    c. How confident are you in conducting initiation services?
    d. How confident are you in conducting refill appointments?
    e. How confident are you in following up on missed appointments?
    f. How confident are you in having ongoing relationships with men you counsel?

3. What part of the interventions do men seem the most interested in?
    a. What works best? What connects to men most?
    b. What seems to motivate men most to return to treatment?
    c. What helps men reinitiate the most?
        1. Receiving medication at home?
        2. Counselling?
        3. Receiving a facility navigation?
        4. Less specific activities like: tracing, following-up, being a friendly face at the facility

PROVIDING CARE

4. What tools helped you most when providing the intervention?
    a. Probes: registers, personal notebook, flipchart, talking with colleagues, talking with supervisor

5. What prevents you from providing the intervention well? What do you find most difficult/challenging?
    a. Probes: complicated registers, required use of flipchart, inability to talk with colleagues, inability to talk with supervisor, funds/money, transport?

6. One of the main tools you have used in this study is the flipchart. Think about how you have used the flipchart. Remember there is no right or wrong answer. We want you to be honest so that we know how to make improvements.
    a. How often do you use it?

b. Do you feel it was helpful for you? Why? Why not? *PROBES: It's smaller than the traditional counselling flipchart, it has probes and questions in bold, it has pictures specific to men so you can connect with it too*
c. Do you feel it was helpful for men? Why/what was specifically helpful? Why not? *Probes: pictures specific to men*

7. What else would help you to provide high quality counselling and treatment tailored to men in the future? *(NOTE: this should not be financial, monetary. Focus more on specific tools, trainings)*

8. The counselling material you have been using was adapted to be male-specific. Do you think this meant that the men could connect with the material more than other counselling styles? Or was it just a repeat of the same information for them?

9. In the last focus group, some of you talked about self-reflection and working to improve your counselling. All of us always improve and do better. What do you think you could improve on and how do you think you can do this? Or, if you have already been trying to do this- what have you been doing?

    a. *PROBES: What about elements that were discussed in the refresher training as having room for improvement? E.G. Actionable next-step plans, counselling on little-by-little, expressing empathy to your clients*

10. Do you feel that you, yourself, are open with your clients?
    a. *PROBES: Do you share your own stories with them?*
    b. Why/Why not?
    c. Do you feel like sharing your own stories is a useful tool during counselling?
    d. Are there other strategies that you use for relating to your clients? What are these?

CLIENTS

11. What have you enjoyed the most about working with these men?

12. You have interacted with a wide range of different men. Can you think of anything they had in common? For example, was there a common struggle or experience that they had?
    a. Were there any men whose situation was very different or unique?
    b. What were your impressions of these men- if you could characterise them, how would you? Remember there are no right or wrong answers. *(PROBE with some descriptions like: motivated, lazy, shy, etc.)*

13. *NOTE: Responses could be the same as to question 12, if so prompt for other answers or skip.* What was the most common reason for default among the men you have counselled?
    a. Do you think the counselling helped these men to overcome these issues? Why/why not? If it did not, what else do you think they need? *PROBE: think about the clients that you counselled or tried to counsel and they did not initiate*
    b. Do you think that the different models for care (facility navigation, home-based treatment, male mentorship) helped these men to overcome these issues? Why/why not? If it did not, what else do you think they need? Remember, not everyone is the same- it might have helped many men but there are surely some you have met that still struggle.

14. Think of the men that you have been working with. What do they talk to you about? What issues do they most commonly have, and want to discuss with you? Think about your counselling sessions, and the interactions you have with them afterwards.

15. Is there anything that clients do not want to discuss with you? What?

16. We want to understand how your clients engage with you
    a. How often do you feel you have good conversation (i.e. a back and forth exchange, discussing topics specific to the man himself, not just the materials)?
        1. Never, Not often, Often, Very often, Always
    b. How often do you feel you are just 'talking at' the client (i.e. the client is not very responsive or there is minimal discussion)?
        1. Never, Not often, Often, Very often, Always
    c. How often do you feel that you are able to make tangible next steps with clients for their care? *Note: Next steps that go beyond just telling the client what to do.*
        1. Never, Not often, Often, Very Often, Always.
        2. *PROBE: Are you clients able to actively join in on these discussions or is there something that holds them back from working with you?*

17. What do clients want from you? Based on your experiences with men in the study, you might have ideas about what you think men need in order to stay on ART. What do you feel that men want from healthcare workers like yourselves? *PROBES: Ongoing relationships, medicine brought directly to them etc.*
    a. Do you have suggestions about what could be done differently? If you could change one thing about the healthcare system to help support men, what would you do? *PROBES: HCWs need to be more patient with clients, clinics need to be open later. If answers centre around providing clients with money or transport then try to suggest other options*
    b. Is this unique to men? Or do your thoughts here also apply to non-study clients (women etc.)?

18. Do men you meet generally return to care? This not to assess your performance, this is just to help us understand these men and their lives.
    a. Why?
    b. For those that don't- why? What do you think holds them back?
    c. Do you think these issues are unique to men?

19. We know that (re)starting is just the first step. For men in the studies who return to care, do you think they will manage to continue to take medication and be retained in care for months or years to come? Think about your clients that you have worked with to help you consider your opinion.
    a. Think of those you think will continue: Is there anything about them/their lives/their experiences that makes you think this?
       Think of those you think will struggle to continue: Is there anything about them/their lives/their experiences that makes you think this?

20. What's the most difficult thing for men when starting ART?
    a. Do you think this is unique to men/study clients?

21. We know some of these men experience unique struggles. Have any of the men you've had in the study experienced adverse events? *PROBES: IPV, unwanted disclosure, depression, substance abuse, acute illness.* Please remember, you should not share client names.
    a. What could be done to help these men with these issues?
    b. What could be done to support you as HCWs when helping men with these issues?

22. We know that at times you have had a unique relationship with the men you have been counselling. Is your relationship with them different to how it is with other HCWs? How? Why?

FINAL THOUGHTS

23. Do you have any final thoughts to share? Something that you have experienced that might be unique, something that you think your colleagues could benefit from hearing, something that you think would support you as HCWs in the future?
