# Supplementary figures and images for: Core components of male-specific person-centred HIV care: a qualitative analysis from client and healthcare worker perspectives in Malawi
Source: BMJ Public Health. 2024 Dec 22;2(2):e001100. doi: 10.1136/bmjph-2024-001100 (PMC11816952; doi:10.1136/bmjph-2024-001100)

#### Supplemental Information 4: Male-specific Person Centered Care Job Aid for Programmatic Use

---

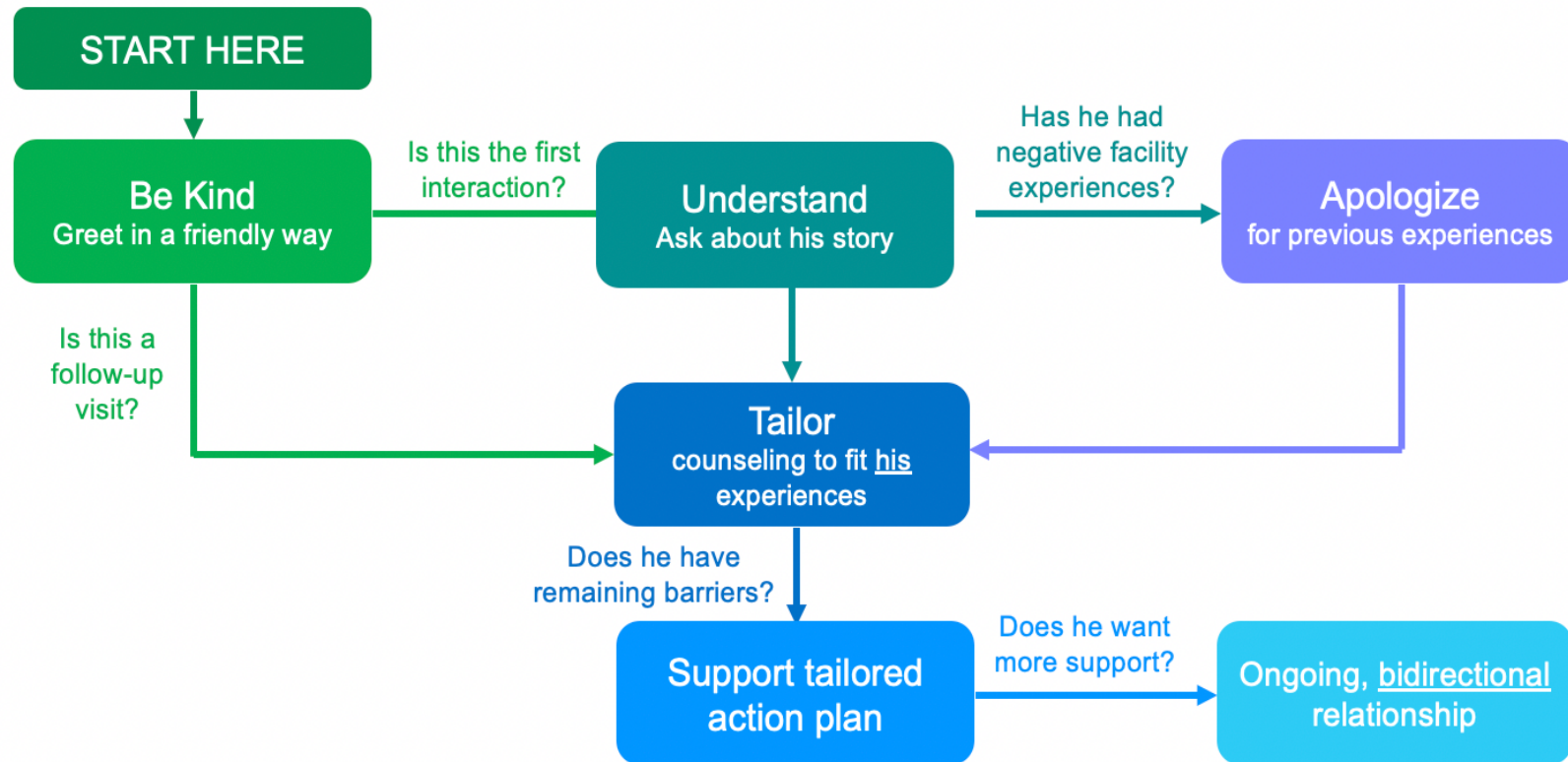

Supplement: online supplemental file 4 [file bmjph-2-2-s004.pdf]
